# A Novel Model-Based Driving Behavior Recognition System Using Motion Sensors

**DOI:** 10.3390/s16101746

**Published:** 2016-10-20

**Authors:** Minglin Wu, Sheng Zhang, Yuhan Dong

**Affiliations:** Advanced Sensor and Integrated System Lab, Graduate School at Shenzhen, Tsinghua University, Shenzhen 518055, China; wml15@mails.tsinghua.edu.cn (M.W.); dongyuhan@sz.tsinghua.edu.cn (Y.D.)

**Keywords:** driving behavior recognition, physical model, data change rule, motion sensors, Kalman filter, adaptive time window, classification

## Abstract

In this article, a novel driving behavior recognition system based on a specific physical model and motion sensory data is developed to promote traffic safety. Based on the theory of rigid body kinematics, we build a specific physical model to reveal the data change rule during the vehicle moving process. In this work, we adopt a nine-axis motion sensor including a three-axis accelerometer, a three-axis gyroscope and a three-axis magnetometer, and apply a Kalman filter for noise elimination and an adaptive time window for data extraction. Based on the feature extraction guided by the built physical model, various classifiers are accomplished to recognize different driving behaviors. Leveraging the system, normal driving behaviors (such as accelerating, braking, lane changing and turning with caution) and aggressive driving behaviors (such as accelerating, braking, lane changing and turning with a sudden) can be classified with a high accuracy of 93.25%. Compared with traditional driving behavior recognition methods using machine learning only, the proposed system possesses a solid theoretical basis, performs better and has good prospects.

## 1. Introduction

With rapid economic development, vehicle ownership worldwide has been increasing in recent years. Unfortunately, in addition to increasingly severe road congestion, the growing number of vehicles is posing a threat to traffic safety and social security. Statistics released by the Ministry of Public Security show that there existed 283 million vehicles and 335 million drivers in China at the end of March 2016 [1]. What is worse, about 60 thousand people die and over 200 thousand people get wounded in traffic accidents every year and more than ninety percent of fatal accidents are caused by offensive driving behavior [2]. Under some circumstances, cautious drivers may have to suffer as a consequence of the actions of those who do not take the responsibility of driving seriously and even those who engage in aggressive behaviors for thrill-seeking purposes. To admonish aggressive drivers and eliminate this phenomenon, many articles [3,4,5,6,7] have discussed and emphasized the recognition of typical driving behaviors. Apparently, recognizing drivers’ behaviors (including normal driving behaviors and aggressive driving behaviors), recording their driving patterns and feeding information on their driving behaviors back to themselves or relevant departments can help to promote safer driving, reduce traffic accidents and contribute to social safety.

Automobile manufacturers are installing advanced driver assistance systems (ADAS) in some high-end cars with the purpose of balancing safety and efficiency among road traffic [8]. The ADAS mainly consists of an electronic stability program (ESP), an adaptive cruise control system, and a lane departure warning (LDW) system [9]. The relatively high cost of each part limits the deployment of ADAS in economical cars which constitute the majority of cars on the road. In addition, the ADAS mainly concentrates on driving assistance rather than evaluating driving style.

Similar to ADAS, utilizing hybrid signals (including signals sampled from video, microphone, GPS, pressure and inertial sensors, and etc.), authors in [10,11] discussed and summarized several approaches to recognize driving behaviors and monitor drivers. When being taken as inputs of driving behavior recognition systems [5,7], however, these signals are difficult to transmit and process, which increases computing complexity and affects system automation. Consequently, the utilization of these information can be difficult to some extent [12]. What is more, it has been proved that the output signals by inertial sensors can fully meet the requirements of recognizing different driving behaviors [3,4,6,12,13,14].

Generally speaking, there are two different ways for researchers to obtain dynamic information about moving vehicles: the Controller Area Network (CAN) bus and micro electro mechanical system (MEMS). A CAN bus is a standard vehicular bus to facilitate communication between microcontrollers and devices without a host computer [15], and it carries all the necessary data to describe the state of a car [13]. Through the On-Board Diagnostic (OBD) port of vehicles, people can obtain very limited access to the information of CAN bus. In recent years, MEMS has been fast growing in popularity, becoming smaller in size, lighter in weight and lower in energy consumption, which makes it possible to integrate different sensor units such as accelerometers, gyroscopes, magnetometers, temperature transmitters, GPS, and so on. Mounted in the cab, the MEMS system can provide people with an approach to obtain information on the moving car [13]. Motion sensors and smart phones are among the typical applications of MEMS systems.

Though a CAN bus carries all the vehicle signals, the information people can extract through an OBD port depends on the mastery of private protocol of the particular types of cars. With concerns for safety, automobile manufacturers hesitate to release the private protocol of OBD ports to the public, mainly in consideration of intellectual property and vehicle security. In addition, some researches have revealed that many vehicle components, can be accessed through OBD [16,17], which leaves potential for criminals to control the vehicle. Consequently, OBD products pose a threat to the vehicle and human security to some extent. Furthermore, the work of [13], using the CAN bus data, generated a worse performance than that of a portable device. Therefore, in the process of driving behavior recognition, the data collection platform is more appropriate based on the MEMS system rather than the OBD port and CAN bus.

With the proliferation of smart phones, exploiting the resources of smart phones has become increasingly popular. In some cases, researchers utilized smart phones as sensor platforms and presented some satisfying results [4,7,18]. Though smart phones and motion sensors are all integrated with MEMS systems, they perform differently in terms of driving behavior recognition. Smart phones are embedded with abundant sensors, largely providing better human-device interaction. When more computing resources are allocated to driving behavior recognition, smart phones may experience lag which may result in negative user experience. Also, different types of smart phones may be equipped with different MEMS systems and, as such, perform quite differently. Furthermore, drivers use the smartphones mainly for navigation and communication when driving, which can interrupt recognition and feedback processes within the system. Utilizing motion sensors can avoid such situations since the integrated three-axis accelerometer, three-axis gyroscope and three-axis magnetometer are sufficient to identify different driving behaviors without redundant sensors occupying more processor resources. Compared with smart phones, the motion sensors are mostly low-cost, perform more professionally and attain higher accuracy in the process of driving behavior recognition.

During motion, a vehicle can be treated as a rigid body. Then, the relevant theory relating to rigid body dynamics can be used to explain the change rule of the data (acceleration, angular velocity and magnetic induction intensity) collected by the motion sensors.

In the prior works aiming at recognizing driving events and driving styles, researchers adopted machine learning methods purely to extract data features and deal with classification [3,7,12,13]. However, all these works ignore the theoretical analysis and seem to be done in a black box, which obstructs the further optimization and research of systems. Our work successfully solves the problem above by establishing a physical model to depict a moving vehicle and reveal the data change of each axis. Innovatively, we utilize this physical model throughout all stages of our proposed system, removing noise from data using a Kalman filter, extracting valid data using an adaptive time window, extracting effective data features and classifying data using various classifiers (support vector machine, Bayes network, and so on). The physical model ensures good performance.

In summary, our work makes the following contributions:

- We propose a novel model-based driving behavior recognition system using motion sensors.
- We build a physical model to describe the car moving process and reveal the change rule of the data collected by the motion sensors including a three-axis accelerometer, a three-axis gyroscope and a three-axis magnetometer.
- Based on the physical model built, differently from the prior research, we eliminate the noise from data using a Kalman filter.
- Based on the new physical model, we propose a novel and effective method to extract the valid data from the car moving process utilizing an adaptive time window.
- Based on the new physical model, we extract features from the valid data to prepare for the classification.
- Based on the built physical model, we classify and recognize different driving behaviors using statistics learning methods. The performance of different classifiers is analyzed and the best one is chosen.

The rest of this article is organized as follows. Section 2 introduces the related works in recognition of driving behaviors or driving styles. Section 3 presents the experimental environment and provides a systematic overview of our work. In Section 4, we present a new physical model which depicts the car moving process and explains the data change of each axis. In Section 5, we introduce the Kalman filter to deal with noise elimination and propose a novel method to extract valid data using an adaptive time window as well as feature extraction from valid data based on the new physical model. The results of driving behavior recognition, the performance of various classifiers and the evaluation of the system are reported in Section 6. Conclusions and future works are discussed in the last section.

## 2. Related Work

Undoubtedly, driving behavior recognition systems can help drivers to be more cautious and further contribute to traffic and social safety. There are many researchers devoting themselves to study of relevant areas, such as driving style recognition, driver classification, safe driving, and so on. Based on the research methods used, these studies could be divided into two categories, one focusing on machine learning algorithms and the other utilizing other methods.

### 2.1. Work Related to Machine Learning Algorithms

Mitrovic developed a driving events recognition system using hidden Markov models (HMMs) using the data of longitudinal and lateral acceleration [3]. The limited information carried by the acceleration does not possess the capacity to describe the data change completely in the process of car motion. That is the reason why this work can only recognize seven different sub-typical kinds of driving events without having a better performance in terms of accuracy. B. Higgs and M. Abbas defined the driver behavior as a map function with current traffic state as the argument and driver action as the dependent variable [19]. This work utilized segmentation and clustering to decompose the single map function in traditional car-following models into several different functions. Based on these functions, researchers defined and evaluated driving patterns of drivers. Johnson et al. proposed a MIROAD system aiming at recognizing driving style using the sensors built-in smartphones [7]. Utilizing data fusion of an accelerometer, gyroscope, magnetometer, GPS and video, based on the Dynamic Time Warping (DTW) algorithm and the K-Nearest Neighbors (k-NN) algorithm, the MIROAD system can recognize aggressive and non-aggressive driving behaviors. Sathyanarayana et al. [13] made a comparison between CAN bus signals and sensory signals from a portable device and concluded that when it comes to driving maneuver recognition, there was a 15% improvement using data collected by a portable device. In addition, this work discussed the choice of features and compared the performance of k-NN and SVM. It has been proved that utilization of sensory information from a portable device is better than the CAN bus data obtained through an OBD port in the domain of driving behavior recognition. In [20], Wang et al. summarized the three methods to recognize and predict driving conditions: the GPS based technique, statistic and clustering analysis and Markov chain. Based on the pressure information concerning acceleration and brake pedal, the authors also categorized drivers according to three driving styles which were mild, normal and aggressive. These works have been done to obtain optimal fuel economy for hybrid electric vehicles.

### 2.2. Work Related to Other Methods

An international collaborative team led by Takeda et al. equipped three vehicles with numerous sensors including cameras, microphones, OBD devices, GPS, inertial sensors and pressure sensors, etc. [10]. These three data sampling and transmission platforms can be utilized to monitor drivers and accurately determine the status of the moving car, which effectively offered research support concerning driving behavior. Vaitkus et al. [6] proposed a pattern recognition approach to evaluate normal and aggressive driving styles using statistical data of a three-axis accelerometer, and discussed the feature extraction in the time domain, but failed to incorporate the recognition of driving events in their work. Fazeen et al. [4] utilized three-axis accelerometer data of an Android-based smartphone to record and analyze driver behaviors including accelerating, decelerating and changing lanes. However, this work did not classify the specific driving behaviors. Emphasizing recognizing road conditions, researchers identified road anomalies and mapped experimental road segments. Doerr et al. [21] developed an online driving style recognition system using fuzzy logic. Instead of collecting data in real traffic conditions, the authors utilized the platform *CarMaker* from *IPG* to build a so-called *vehicle control model* to generate data depicting car motion. Also, this work simulated CAN bus signals, which would have some restrictions for practical application. Aimed at analyzing psycho-physiological states of drivers, Rygula [22] developed a new driving style identification method which uses tachograph for speed analysis. Dai et al. [14] utilized the information extracted from accelerometer and orientation sensors integrated in smartphones to detect drunk driving. Based on their algorithm, the authors compared sampled data with typical drunk driving samples to judge the driving state of drivers. An app for iPhones was developed in [5]. With the computer vision and pattern recognition methods, Bergasa et al. utilized hybrid signals (including video, voice, GPS and inertial sensors) to detect driver inattention. The app would feedback the calculated outcomes to the driver and evaluate the driving process.

Compared with all these related prior works, our system utilizes the fusion of three-axis accelerometer, three-axis gyroscope and three-axis magnetometer data, which has been validated to be sufficient but not redundant. Providing the system with a valid theoretical foundation to ensure it is well-structured and well performed, we further build a physical model to depict the car moving process and explain the data change of each axis. Then, under the guidance of the proposed physical model, we eliminate noise from raw data, extract valid data segments using an adaptive time window, extract data features and classify driving behaviors.

## 3. System Description

The proposed system consists of three components: the hardware part contains motion sensors and serves as the acquisition platform, the software part is designed to handle the acquired data, and the theoretical physical model provides guidance for the whole system.

The motion sensor platform used in our system is iNEMO V2 developed by STMicroelectronics, and the data collector will be set in different cars. The sensors sample the data at a frequency of 50 Hz. CUP module carried on this platform undertakes the task of computing and scheduling resources. The data acquisition is carried out within the iNEMO platform automatically, without using the resources of other devices. In addition, the data collected in the experiment is stored in SD cards and utilized for the research only, without compromising driver privacy and car safety.

The software part consists of the following components: data filtering module, adaptive data processing module and pattern recognition and classification module. Specifically, data filtering module shoulders the responsibility of eliminating noise from raw data. The adaptive data processing module is in charge of extracting valid data and then the feature extraction from it. Pattern recognition and classification module puts extracted features into classifiers to recognize different driving behaviors.

The theoretical support part combines the basic theory of rigid-body dynamics and the analysis of car in motion to build a physical model, deduce the data change rule of different driving behaviors and provide a theoretical basis for the system.

We devote ourselves to establishing a specialized, stable and credible driving behavior recognition system.

### 3.1. Experimental Setup and Environment

**Figure 1:**
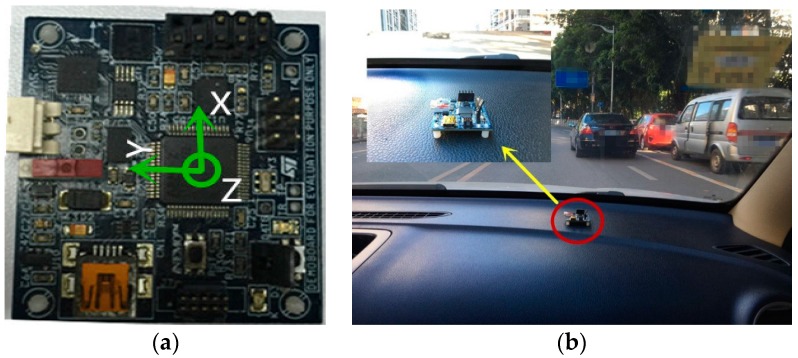
Setup of the board. (**a**) The axis pointing of motion sensor; (**b**) The placement of the board in the car.

In the data acquisition process, the placement of the motion sensors, mainly the rotation and angle, will influence the outcome of the recognition system. Specific to this experiment, the motion sensor is placed on the dashboard, parallel to the ground with its *X*-axis pointing to the car moving direction, the *Y*-axis coinciding with the car’s lateral direction, pointing to the left side, and the *Z*-axis being vertically upward. Figure 1a shows the axis pointing and Figure 1b displays the placement of the board. The reference coordinate system is the ENU (east, north and up) coordinate system.

**Figure 2:**
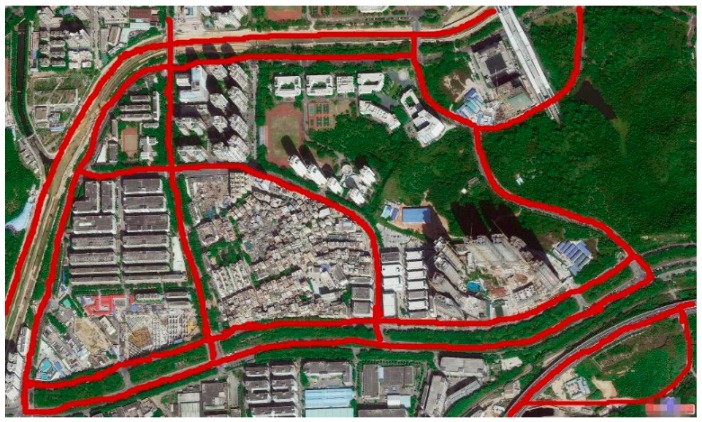
Driving map depicts a part of the covered routes in the data collection process.

In the process of driving, the total drive time is up to 20 h and driving mileage is over 1200 km, covering many roads in Shenzhen, China. In the future research, the driving time and mileage will be substantially longer. The route map covering part of the routes taken in the data collection process is displayed in Figure 2.

In the data collection process, cars move along the different roads marked in red as shown in Figure 2. Without the restriction of time, speed and destination, on the premise of observing traffic laws, drivers can maneuver cars according to their own desires. Five drivers and five different cars contribute to the obtainment of the original data. The information about cars used is given in Table 1.

These cars are used repeatedly and provide the corresponding datasets. Though being small in quantity, these five cars could represent most types of family cars and taxis on the market. In addition, for the driving behavior recognition system, we can consider the moving car as a rigid body or a particle. At the macroscopic level, the physical processes of moving cars are coincident. The data acquired by motion sensors is concerned only with car motion. Consequently, for different types of cars, motion sensors (the data acquisition platform) are universal.

### 3.2. System Overview

The proposed driving behavior recognition system is mainly composed of the following six segments, which form the research process.

1. Based on the theory of rigid body kinematics, we analyze the relationship between acceleration and linear velocity and that between linear velocity and angular velocity. Following the analysis concerning magnetic induction intensity variation, we build the physical model to depict the car moving process and reveal the change rule of data. We prepare a theoretical basis for the whole system.
2. We set up the motion sensor as shown in Figure 1 to gather nine-axis data of different motion states. We expand and consummate the database constantly.
3. We analyze the components and characteristics of noise within the motion data and compare the performance in eliminating clutter of the low pass and Kalman filters. Based on the physical model built, we choose a more appropriate filter to avoid the negative effects on classification and recognition of driving behaviors.
4. Based on the new physical model, we extract the valid data (data segment denoting the driving behaviors, such as accelerating, turning and so on) from the long-time and irregular driving process. Specifically, we leverage the adaptive time window and the novel proposed methods to detect the start and end of valid data. The whole data is partitioned automatically, avoiding labelling data manually, which is more economical and efficient.
5. We extract the data feature vectors, mainly consisting of mean value, peak value, covariance and so on, from the valid data. Guided by the physical model or not, the performances of different feature sets are compared according to the criterion of classification accuracy. We choose the suitable features and prepare for the next step.
6. We analyze the application range and characteristics of different statistical learning methods. Then, we employ various classifiers to classify diverse driving behaviors. We analyze and evaluate the performance of different classifiers and choose the best one.

**Figure 3:**
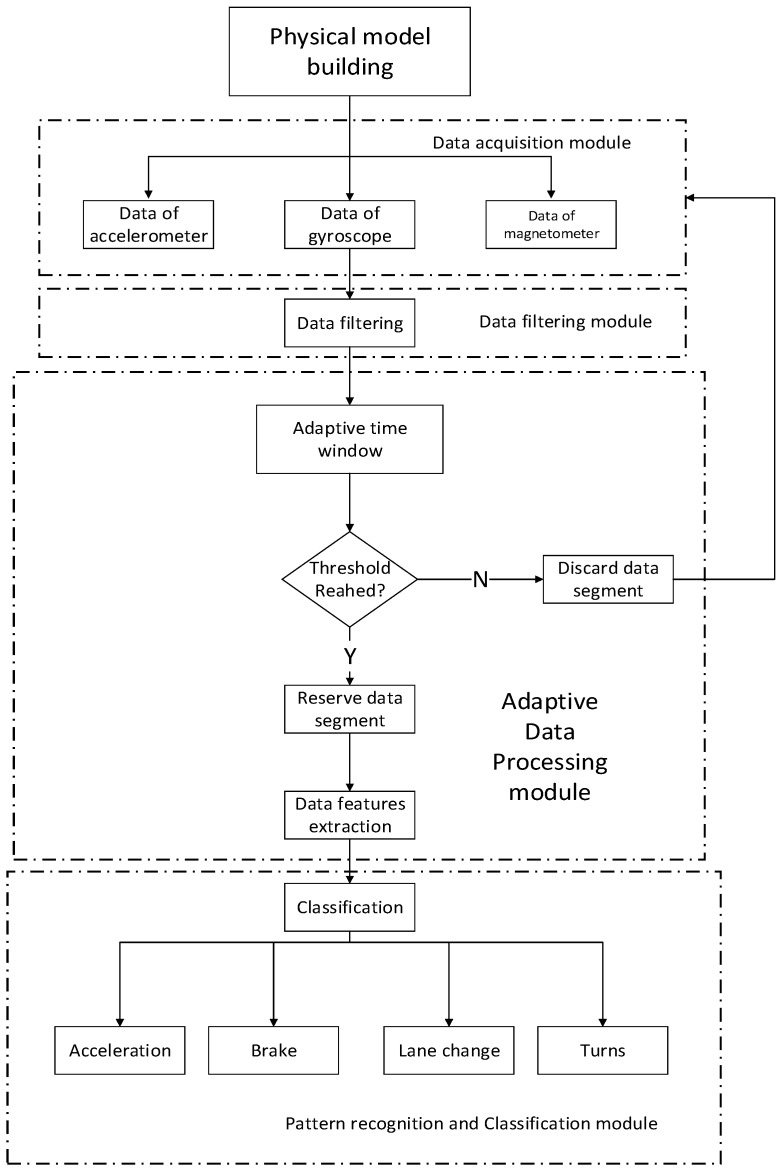
Architecture of proposed driving behavior recognition system.

Figure 3 illustrates the architecture of the proposed driving behavior recognition system.

Ignoring the influence of bad weather, the black spots can be classified as follows: long straight roads, roads with small curve radius and the linear combination of various conditions [23]. Long straight roads make drivers sleepy and cause visual fatigue, which induces unconscious acceleration and lane change. Similarly, roads with small curve radius are often accompanied by braking and turning. Corresponding to the third case, a combination of different driving behaviors can always be observed. Considering the factors above, we choose the driving behaviors listed in Table 2 for the task of recognition.

Generally, driving behaviors to be detected are divided into two categories: normal behaviors and aggressive behaviors. So far, we consider seven specific maneuvers in each category and plan to increase the number gradually in our future work. Driving behaviors in Table 2 cover the most typical ones generated by different drivers in real traffic conditions. By recognizing these behaviors, we can persuasively evaluate driving habits and styles of drivers and construct their archives, which is important in promoting cautious driving.

## 4. Establishment of Physical Model

The works [24,25,26] thoroughly studied the dynamic model or the state observation of vehicles. Relevant knowledge such as vehicle dynamics, different tire models, side-slip angels, velocity and acceleration is incorporated. In the area of driving behavior recognition, however, some of this knowledge can be redundant. When recognizing driving behaviors, we can take a moving car as a unit and ignore its inner structure. The theory of rigid body kinematics is adopted to establish the physical model.

When vehicles are moving in real traffic conditions, their main behaviors include going straight, changing lanes and making turns. In the process mentioned above, acceleration and angular velocity of vehicles change according to certain rules of rigid body kinematics. Specifically, the relationship between acceleration and angular velocity can be represented as follows

(1) $$\vec{v_{t}}=\vec{v_{0}}+\vec{a}t$$

(2) $$\vec{v}=\vec{w}\times \vec{r}$$

In the time domain, $\vec{a}$ and $\vec{w}$ can be decomposed separately into $a_{x},\;a_{y},\;a_{z}$ and $w_{x},\;w_{y},\;w_{z}$.

**Figure 4:**
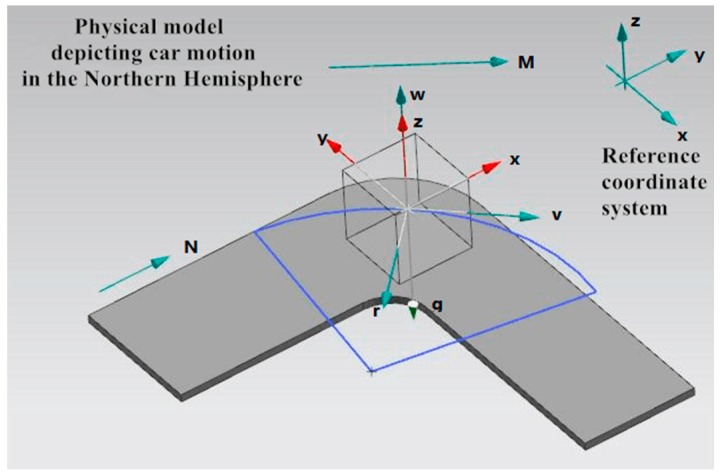
Physical model depicting car motion in the Northern Hemisphere.

Furthermore, the variation of magnetic induction intensity information measured by the motion sensors is not only related to the vehicle behaviors but also connected with its geographic position. The distribution of the geomagnetic field decides the intensity and direction of all places on the earth. Similarly, it can also be divided into $m_{x},\;m_{y}\;\mathrm{and}\;m_{z}$. To account for the change rule of data systematically, we, first and foremost, develop the physical model. Taking the Northern Hemisphere, for instance, the physical model is portrayed in Figure 4. The cube in Figure 4 represents a moving car. The red three-dimensional system of coordinates is set inside the motion sensor and the arrow marked with *N* points to the north. The two arrows with *g* and *M* represent the gravitational acceleration and geomagnetic field, respectively. Take RT for instance, the car moves along the blue arc with turning radius of *r*. Its linear velocity (the arrow marked with *v*) points to tangent direction of circular arc and according to Equation (2), the angular velocity is vertically upward (the arrow marked with *w*). In addition, the geomagnetic field has the downward direction in the area north of the equator. The magnetic field quantified by sensors will vary with the car in motion.

**Figure 5:**
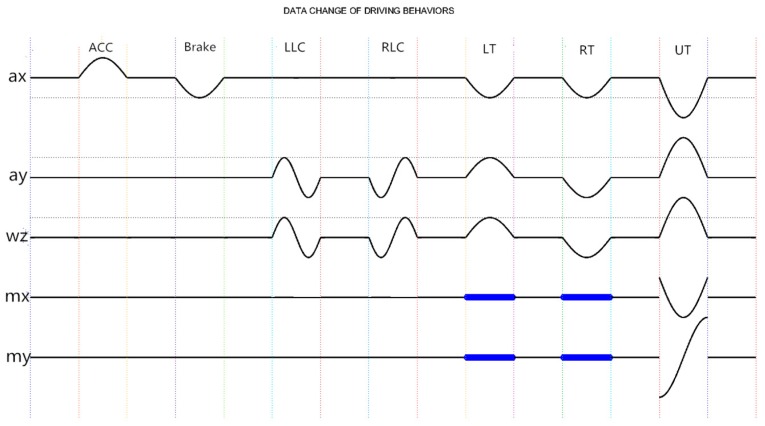
Data change rule of different driving behaviors.

Based on the physical model we built and Equations (1) and (2), the change of data on behalf of different driving behaviors can be deduced and the outcome is demonstrated in Figure 5. Motion sensor is set up as described in Section 3. Furthermore, in the process of moving, five of the nine axes show data variation, which are $a_{x},\;a_{y},$ $w_{z}$, $m_{x}\;\mathrm{and}\;m_{y}$. The waveform in Figure 5 reveals the corresponding data change rule of specific driving behavior.

Apparently, different driving behaviors bring about data changes of different axes. When going straight, car movements such as acceleration and brake possess the strong pertinence with acceleration information, independent of angular velocity. Other behaviors such as turning and lane changing will also cover the information concerning angular velocity. Take the brake and U turn for a more specific example. When braking, according to Equation (1), radial acceleration ($a_{x}$) of a car reduces to a negative value from zero and then returns to zero again. Meanwhile, detected magnetic field varies with the geographic position and other axes remain invariable. When making a U turn at a west-to-east direction, the radial acceleration of a car changes conformably with the brake while lateral acceleration ($a_{y}$) varies oppositely from the X-axis data. According to Equation (2), the changing tendency of *Z*-axis angular velocity ($w_{z}$) is consistent with lateral acceleration. The geomagnetic field radiates from south to north and its latitudinal component is very close to zero. After the process of making a U-turn, the *X*-axis magnetic induction intensity ($m_{x}$) of the car basically returns to the original value acquired at the start of the U-turn and the vector $m_{y}$ points to the opposite direction with the modulus remaining unchanged. Similarly, the output of magnetometer is dependent on the geographic position. The rest axes are not involved in the U turn behavior. It should be pointed out that when going straight, changing lanes and making turns (excluding U turns), data change concerning magnetic field is chaotic and does not follow a uniform rule. Consequently, the magnetic field data is obscured (the blue segments in Figure 5) in the process of establishing a physical model.

Without regard to the data change rules governing driving behaviors, the previous works [3,7,12,13] sent data features into the classifiers for training and classification purposes. Though the performance was barely satisfactory, no discussion of the classification basis has been provided in detail, which comprises the system’s logicality and integrality.

## 5. Model-Based Data Processing

### 5.1. Original Data and Noise Elimination

Plenty of typical samples of driving behaviors have been obtained in real traffic conditions, which can be used to preliminarily verify the correctness and rationality of the established physical model.

**Figure 6:**
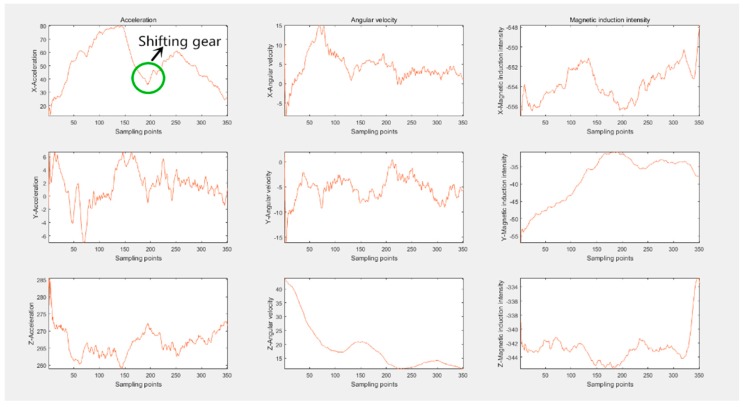
The actual 9-axis data portraying ACC.

**Figure 7:**
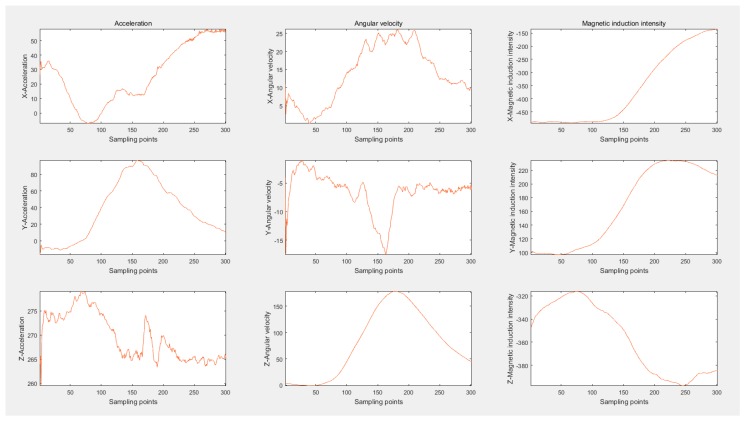
The actual nine-axis data portraying LT.

Given the space constraints, ACC and LT would be taken as examples to show the actual raw data. The data change of ACC is displayed in Figure 6. Among all nine axes, only $a_{x}$ suffers a large change. When accelerating, the longitudinal acceleration of the car will first increase and then decrease. An upwards convex main lobe and a side lobe resulting from shifting gears form the accelerating process of cars. Correspondingly, without shifting manually, the automatic cars complete the ACC just with an upward convex wave. Compared to the change extent of $a_{x}$, the variation of the remaining eight axes can be ignored. Figure 7 portrays data change of LT. The $a_{x}$ of LT shapes a wave trough to decrease the longitudinal velocity, while $a_{y}$ and $w_{z}$ form a crest, respectively, which alters the orientation of velocity. Conforming to the theory of data change rule in Section 4, the actual data depicting driving behaviors proves the correctness and rationality of the proposed physical model.

**Figure 8:**
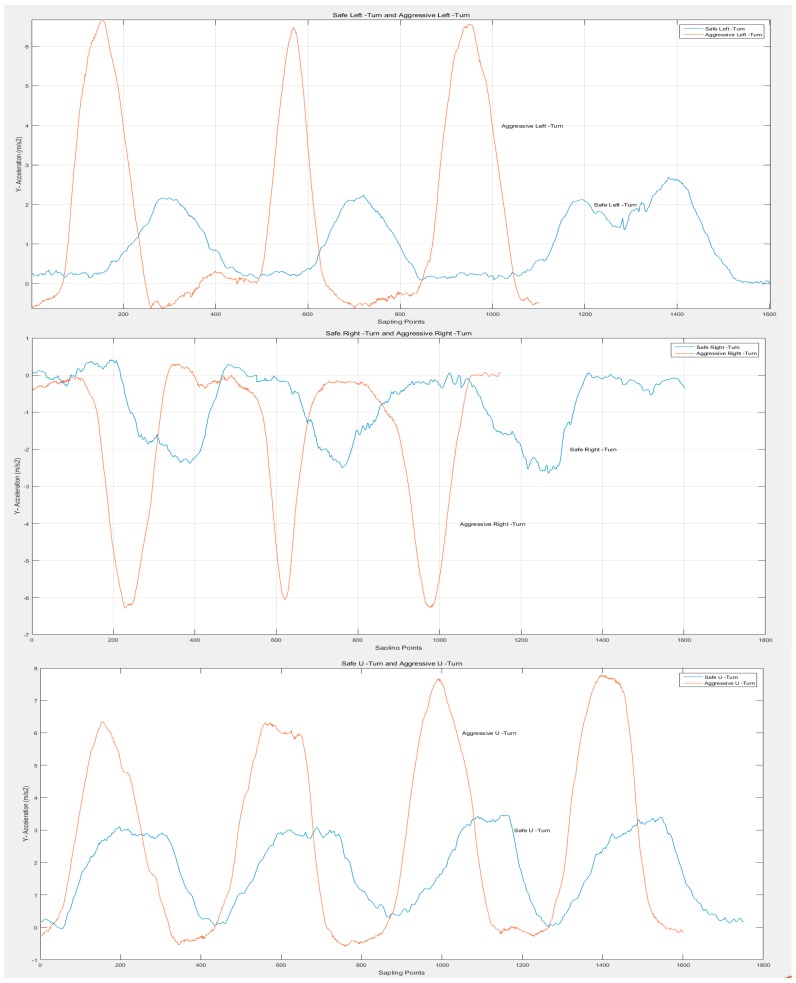
Data comparison of *Y*-axis acceleration between normal and aggressive LT, RT and UT.

As mentioned earlier, driving behaviors can be classified into normal and aggressive types. Since aggressive driving behaviors can greatly compromise traffic safety, we collected some samples of these behaviors for recognition. Though the data change rule of aggressive driving behaviors abides by what is described in Figure 5, there exists a significant difference between the normal and aggressive behaviors. The data comparison is demonstrated in Figure 8, citing $a_{y}$ of LT, RT and UT.

The blue curves in Figure 8 denote LT, RT and UT while the red ones describe A-LT, A-RT and A-UT. Apparently, the waveforms of aggressive behaviors suffer a sharper increase and decrease, a shorter duration and a larger domain for amplitude change. For instance, when turning left aggressively, cars can produce an $a_{y}$ over 6 $m/s^{2}$, in contrast to the 2 $m/s^{2}$ of normal LT. These obvious differences provide the slope of data waves, the peak value and the duration of data change to facilitate the classification of normal and aggressive driving behaviors. The recognition of aggressive driving behaviors can contribute to evaluating the driving style of drivers and improve their driving by feeding the information of aggressive behaviors back in a timely manner.

In the moving process, the transmission for the real data generated from a car to the output of the motion sensor can be considered as a linear system. Inevitably, data acquisition of motion sensors will be disturbed by various factors, such as traffic congestion and different noises. The stop-and-go or bumper-to-bumper traffic conditions make vehicles move very slowly and reduce traffic risks to some extent. So, ignoring the influence above will not compromise the recognition of driving behaviors. The noises in data are mainly composed of vibration noise and white Gaussian noise. Apparently, motion sensors will always quiver in the car and speed bumps and uneven roads will also bring vibration, which affects the sampling process and introduces vibration noise. These brief and frequent vibrations of motion sensors introduce a random offset to the output and make the noise distribution similar to that of white Gaussian noise. Other noise components such as thermal noise and shot noise generally belong to the category of white Gaussian noise.

To achieve a higher recognition rate, the noise must be eliminated as much as possible. Under the guidance of the new physical model, we can evaluate the performances of different filters according to the data change rule shown in Figure 5. In this proposed system, different from the traditional method utilizing the low pass (LP) filter in the area of driving behavior recognition, we apply the Kalman filter to remove the noise and interference from data. Kalman filter is applicable for linear systems and can perform well in eliminating Gaussian noise. Note that the noise successfully removed by the Kalman filter is not necessarily Gaussian [27]. The prediction is that Kalman filter can promise a good performance in eliminating vibration noises and white Gaussian noises.

**Figure 9:**
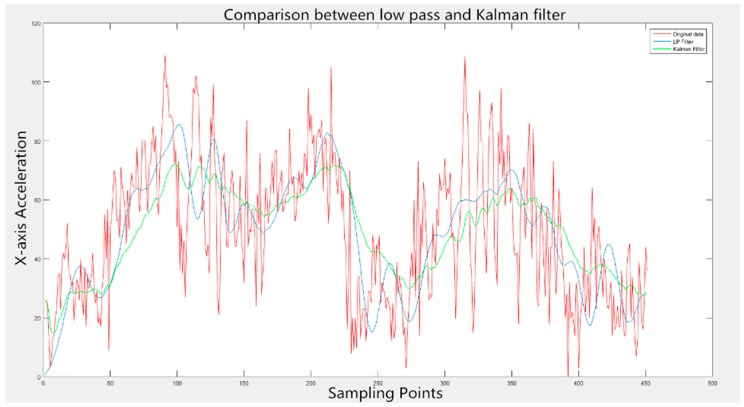
The performance of low pass and Kalman filters.

**Figure 10:**
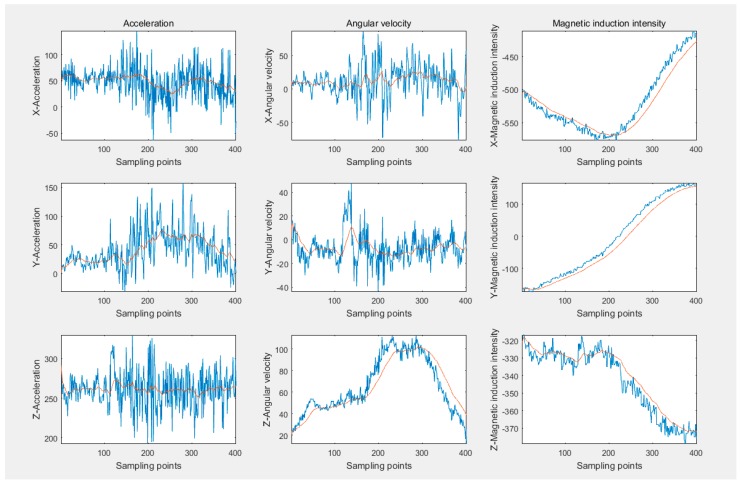
The performance of Kalman filter in filtering all nine-axis signals.

The performance of Kalman and low pass filters is compared in Figure 9. The red curve depicts original data acquired, with the blue one being the outcome of the LP filter and the green one representing the result of the Kalman filter. Obviously, though the whole changing tendency could be recognized, the original data is accompanied by overwhelming noise and the local changes are totally divergent from the data change rule. The LP filter possesses a quicker response to the original data, which causes the noises to exert more influence on data filtering and interferes with the smoothness of waveforms. Fortunately, the prediction-based Kalman filter can avoid the disadvantages mentioned above. When most noises are removed, the output data reserves most of the information contained in the original data and more consistently meets the data change rule in Figure 5, sufficiently enough to complete the subsequent work. Therefore, the Kalman filter is utilized to eliminate noises with the filtering effects of nine-axis signals displayed in Figure 10. The blue curves represent original data and the red ones demonstrate the filtered data.

### 5.2. Valid Data Extraction Using Adaptive Time Window

In the process of driving in real traffic conditions, typical driving behaviors only account for a small portion of the time the car is moving at a basically constant speed. From the physical model and data change rule shown in Figure 4 and Figure 5, we can conclude that various driving behaviors possess corresponding data waveforms. Also, when moving at a constant velocity, the acceleration and angular velocity of a car remain unchanged. Utilizing the slope, shape of waveform and the energy information resulting from data change, we can select the valid data. Given that the durations of different behaviors differ, an adaptive time window is used. The adaptive time window is composed of an indefinite number of basic time windows, the length of which is decided by the sample frequency of inertial sensors used. In our work, we set a basic time window with a duration of 0.2 s and covering 10 sampling points. The basic time window is slid to detect the beginning and end of valid data. Specifically, we develop the following three discriminate ways to judge whether the data in the basic time window indicates driving behaviors or not.

● Gradient (slope) discrimination

As depicted in Figure 5, when driving behaviors occur, the gradient of the data sequence will change. Calculate the absolute value of gradient in the basic time window as follows

(3) $$G=\left|\frac{\left(d\left(x+k-1\right)-d\left(x\right)\right)}{k-2\times \left(sp-1\right)}\right|$$

where k is the number of sampling points in the basic time window. To avoid the interference of a singular value, the mean value of the first and last sp points in the sliding window are calculated to represent the endpoints. And d(x+k−1) and d(x) respectively represent the calculated endpoints.

From Figure 6, we can see that just as $a_{x}$, the data of other axes also suffers a smaller variation in the process of ACC. The variations will generate a gradient change and then affect the extraction of valid data. To avoid this effect, appropriate upper threshold $G_{t}$ is set after analyzing the collected typical samples. We reserve the data of this basic time window temporarily when the calculated G exceeds $G_{t}$, or send the data for other judgments otherwise.

● Comparison between mean value and endpoints

Calculate the mean value of data in the basic time window, recorded as m. For the purpose of avoiding a singular point, we compute the mean value of the first and the last three sampling points in the basic time window to represent the value of endpoints, which are recorded separately as $m_{s}$ and $m_{e}$.

If $\left(m>m_{s}\;\&\;\;m>m_{e}\right)$ or $\left(m<m_{s}\;\&\;\;m<m_{e}\right)$, the data is reserved temporarily. Otherwise, the data is sent to other judgments. This comparison is developed to detect the crest and trough of the sampling sequence.

● Energy discrimination

Calculate the energy of data in the basic time window. Taking *X*-axis acceleration for example, the computational formula is given as follows:

(4) $$E=\frac{g_{x}^{2}\left(i\right)+g_{x}^{2}\left(i-1\right)+\ldots +g_{x}^{2}\left(i-k-1\right)}{k}$$

where $g_{x}\left(i\right),\;g_{x}\left(i-1\right),\ldots ,g_{x}\left(i-k-1\right)$ represent all the k sampling points in the basic window. Similar to the gradient discrimination, the upper threshold $E_{t}$ is set by analyzing typical driving behaviors to avoid the interference from small fluctuations of data or generated noise. If the calculated *E* exceeds $E_{t}$, the data of this basic time window is reserved temporarily. Otherwise, the data is sent for other judgments.

**Figure 11:**
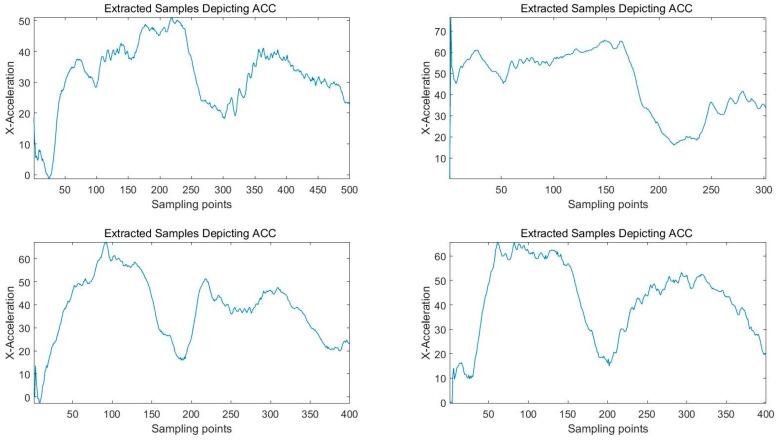
Four samples extracted depicting ACC.

These three different approaches above possess complementary relations. Furthermore, in our work, using these methods in combination can promise us a complete extraction of valid data. If none of the three conditions are satisfied, the data in the basic time window will be abandoned. In order to extract the true valid data, we not only analyze the data of stationary state and that of typical driving behaviors but also take into account the effects of various noises. According to Equation (4), the minimum threshold $E_{\min}$ is set. Even though the data in basic time window has met at least one condition mentioned, only when the calculated *E* is greater than $E_{\min}$, the data can be saved. Then, the valid data can be extracted completely through the detected beginning and end points. What is more, considering realistic conditions, the data can be saved only when the length of valid segments extracted is between 1 s and 15 s. In other cases, abandon the data and continue sliding the basic time window for the next detection. Take $a_{x}$ of ACC for example, the extracted valid data is shown in Figure 11.

### 5.3. Data Features Extraction

The filtered data cannot be treated as direct input of classifiers, for which the extracted data features are just acceptable. Apparently, the classification results depend heavily on the selection of feature sets. However, so far there is no universal standard concerning how to extract or select data features in the area of driving behavior recognition. Figure 5 shows that different driving behaviors possess different data versions. It is this difference that provides the intrinsic basis for driving behavior recognition and offers guidance in extracting data features. Therefore, the information reflecting waveform will be the focus of the feature extraction process.

During accelerating and braking, only $a_{x}$ presents disciplinary change with the former being upwards convex while the latter being upwards concave. $a_{y}$ and $w_{z}$ would also suffer variation for the turning of cars. For an extreme example, utilizing the mean and peak value of $a_{x}$, $a_{y}$ and $w_{z}$ can distinguish acceleration, brake, turns and using the slope information can distinguish left and right lane change. Some examples are listed in Table 3 to illustrate the extreme condition. For instance, we set a suitable threshold ($m_{t})$ of $a_{x}$ to separate ACC and Brake. Utilizing Equation (5), the recognition rate reaches 100%. Similarly, of all the driving behaviors, only the magnetic induction intensity of UT presents regular variation, which can be taken as a feature to help select UTs.

(5) $$Driving\;behavior=\{\begin{array}{c} ACC\;if\;\left(mean\;of\;sample\geq m_{t}\right)\;\\ \;Brake\;if\;(mean\;of\;sample<m_{t})\; \end{array}$$

In addition, it is obvious that the change of *Y*-axis acceleration and *Z*-axis angular velocity is synchronous while that of *X*-axis acceleration and *Z*-axis angular velocity has no obvious relationship. Therefore, the covariance matrix demonstrating the correlation of one axis to another is indispensable in the feature extraction process. What is more, in sharp contrast to normal driving behaviors, the characteristics of aggressive ones in terms of amplitude, duration and slope could also help with classification.

In our work, we extract eleven types of data features in the time domain with descriptions listed in Table 4. Referring to Figure 5, we abandon some obvious redundant items and develop an original feature set. Specifically, a 73-dimensional (5 from max, min and k; 9 from avg, std, peak, pvam, mad and eng; 3 from cov and 1 form t) feature vector is calculated from valid data. Classifying the feature vectors leveraging SVM (Support vector machine) algorithm, the overall accuracy is 91.15%.

Since redundant features would increase computation complexity and waste computing resources, the principle of extracting data features is to utilize the fewest features to contain the most data information. Figure 5 indicates that only five axes ($a_{x},\;a_{y},\;w_{z},\;m_{x},\;m_{y}$) are relevant to the driving behaviors under recognition. To further reduce redundant computation and simplify the feature sets, we only reserve the *mad* defined in Table 4 to portray the basic waveform information of the remaining four axes ($a_{z},\;w_{x},\;w_{y},\;m_{z}$). Specifically, a 53-dimensional (5 from max, min, k, avg, std, peak, pvamand eng; 9 from mad; 3 from cov and 1 form t) feature vector is selected and shown in Table 5. Similarly, by sending them to the SVM, the overall accuracy is increased to 93.25%.

Innovatively, the physical model makes it possible to avoid the traditional approach of selecting the most discriminative features using relevant machine learning methods, such as the liner discriminant analysis (LDA) and the sequential feature selection (SFS) [6,13]. It is obvious that we can greatly save computing resources. What is more, our methods also avoid the empirical selection and optimization of feature sets [3,12]. The fewer features and less computations together with a promise of better performance recommends the direction for further research.

## 6. Results and Analysis

The machine learning methods are introduced for automatic driving behavior recognition. In this section, we present the final results of the proposed system generated by various classifiers.

In our work, SVM algorithm, RBF Network (radial basis function network), Logistic (logistic regression algorithm), BayesNet (Bayesian network), C4.5 decision tree algorithm, k-NN (k-nearest neighbor) algorithm and naïve Bayes algorithm are utilized to complete the recognition process. Take k-NN and SVM algorithms for example. k-NN calculates the distance to find the k nearest neighbors of the testing sample. Then, the testing sample will be assigned to the class that the majority of the k neighbors belongs to. We set k = 4 in our work. SVM is a binary classifier which divides the samples by an optimal separating hyperplane to maximize the margin between support vectors and all possible separating hyperplanes. Furthermore, the one-versus-one classification can be utilized to apply SVM in multi-class problems.

Supporting by the 100% recognition accuracy between ACC and Brake with the mean value information of $a_{x}$ only, Table 3 lists some examples which can be classified even by the information of a single axis. Obviously, the enormous difference between different driving behaviors provides a solid basis for the one-to-one classification and can promise SVM a satisfying performance.

**Figure 12:**
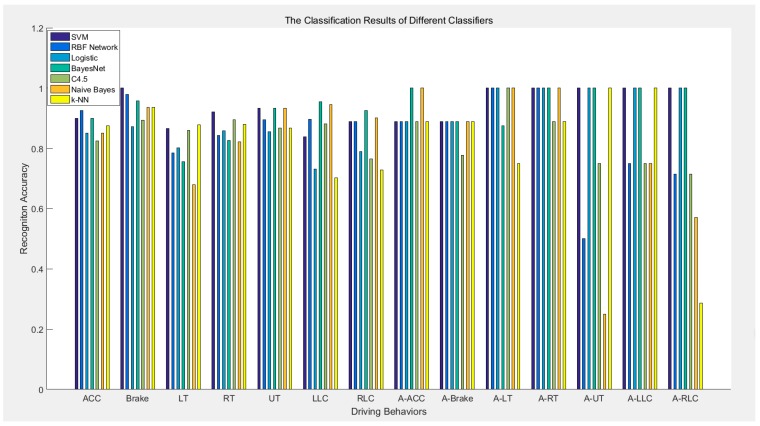
The performances of different classifiers.

In our work, 14 types and a total of 735 driving behaviors are recognized. Specifically, there exist 681 normal and 54 aggressive samples. Using the cars listed in Table 1, all the samples are collected in real traffic conditions. Having eliminated the noises by Kalman filter, extracted valid data using an adaptive time window and extracted feature vectors under the guidance of data change rule, we send processed data to various classifiers. A 10-fold cross-validation is applied to avoid dependency on data. The classification results are demonstrated in Figure 12.

The recognition rates of classifiers vary greatly. The quantitative results indicating the average accuracy of 14 types of driving behaviors for different classifiers are shown in Table 6.

Recognizing 93.25% of the driving behaviors correctly, SVM possesses the best performance among these seven classifiers. As mentioned earlier, the significant difference between each pair of driving behaviors and their separability with several features promote the performance of SVM. Taking into account the independence of different features, which can be proved by the synchronous change of $a_{y}$ and $w_{z}$, BayesNet follows SVM with an accuracy of 91.1%. Logistic achieves a recognition rate of 89.3%, and the RBF Network, C4.5, naïve Bayes and k-NN have relatively poor performances. The recognition results suggest that SVM performs best for such a database and data processing procedure.

Different from conventional methods, we process the data based on the data change rule in every stage, trying to make the samples easy to distinguish. Compared with previous works, the performance of our whole system is convincing. The difference in emphasis and applied algorithms distinguish the recognized catalogue of driving behaviors with a comparison shown in Table 7. The article [3] utilized a low pass filter to reduce noise, a waveform segmentation technique to segment data and HMM (Hidden Markov Model) to classify driving behaviors. This article reached an average accuracy of 91%. Besides the low pass filter, [7] extracted valid data using SMA (simple moving average) of energy and utilized DTW and k-NN to get a 91% recognition rate. In [13], driving behaviors were labelled manually. Then, LDA and SFS were applied to simplify the feature set, and SVM algorithm achieved an accuracy of 89%. Unable to provide a clear idea for further optimization, ignoring the data change rule and just leveraging the knowledge of machine learning can restrict the improvement in system performance. Apparently, benefitting from this solid theoretical support, our recognized catalogue is more substantial with a higher total accuracy of classification. The utilization of a physical model in processing data and classifying advances the system greatly.

In our work, the classification results output by SVM of specific driving behaviors are displayed in Table 8. The normal driving behaviors are composed of 40 ACC, 47 Brake, 172 LT, 191 RT, 76 UT, 74 LLC and 81 RLC. Considering personal safety and traffic safety, we collect fewer aggressive samples. Specifically, there exist 9 A-ACC, 9 A-Brake, 8 A-LT, 9 A-RT, 4 A-UT, 8 A-LLC and 7 A-RLC behaviors. The significant difference between normal and aggressive samples makes them easy to be distinguished.

Among all samples, 4 ACC, 22 LT, 15 RT, 5 UT, 12 LLC, 9 RLC, 1 A-ACC and 1 A-Brake behaviors are wrongly classified. The quantitative recognition rates output by SVM are displayed in Table 9.

Generally, the recognition results can be labelled according to three situations: complete, easy and difficult to distinguish. The Brake, A-LT, A-RT, A-UT, A-LLC and A-RLC belong to the first situation, with all samples recognized correctly. With an accuracy rate of around 90%, the ACC, RT, UT, RLC, A-ACC and A-Brake belong to the second situation. Being hard to distinguish, LT and LLC belong to the third one. By analyzing the recognition results, we summarize that there might exist some factors affecting the performance of classifiers.

● The dataset is not completely ideal.

All the data used in this work is acquired in the real traffic situations and diverse road conditions. When a car drives on the bumpy road segments or through a deceleration strip, its vibrations will introduce chaotic noise to motion sensors. In addition, different drivers have different habits while driving. In most cases, in the process of manipulating the cars, drivers usually apply many redundant operations, such as momentarily shifting gears, accelerating, braking, and so on. These operations will also introduce noise to the motion sensors. Though we utilize the Kalman filter to eliminate noise from data, the fact described above will also cause the data change to deviate from the rule as in Figure 5.

● The traffic flow is complex.

The growing number of vehicles exacerbates road congestion and makes traffic flow more and more complex. In some cases, certain incidents can be counterproductive. For example, even if the drivers are changing lanes to the left, they may brake just to avoid left-hand cars for safety. Consequently, the driving behavior becomes difficult to recognize.

● Similarity exists among different driving behaviors.

The data change pattern depicted in Figure 5 and the actual driving experience all reveal that similarity exists among different driving behaviors. Take the U turn for instance. Vehicles in China are with the left rudder and right line, and the UT mostly turns to the left, making a LT similar to a UT in all data changing trends except $m_{x}$ and $m_{y}$. Consequently, UT can be considered as the combination of two LT to a large extent. It is the main reason why classifiers classify four UTs as LTs and four LTs as UTs. For another example, in some cases, changing lanes is accompanied by the variation of *X*-axis acceleration, which makes LLC or RLC similar to LT or RT. That is why 10 LLC behaviors are classified as LTs improperly and the accuracy is lower than others. What is more, the similarity also causes 12 LTs to be recognized as LLCs, nine RTs as RLCs and seven RLCs as RTs.

● The existence of singular data.

Data acquisition in real traffic conditions may be dangerous. Some behaviors, mainly the aggressive driving maneuvers, cannot be done integrally for the consideration of traffic safety. In addition, in the early stage of analyzing driving data and building physical models, the driving behaviors need to be labelled manually. Because of the limitation in energy and concentration, people may misclassify driving samples, which is unavoidable. That is why singular data exists in the dataset.

● The combination of different driving behaviors.

Different driving behaviors collected in real traffic conditions are not always completely distinguished and independent, which makes the data portray combinative events. The acceleration is often accompanied by turning at traffic lights. Having turned to another direction, driver may adjust the velocity and lanes of the car to gain a better driving experience. The condition above can explain the process of classifying three ACCs as LTs and four RTs as ACCs.

Though there are some factors prejudicing the performance of classifiers, the final accuracy of 93.25% achieved by SVM algorithm is persuasive. The overwhelming majority of samples in the dataset conforms to the data change rule deduced from the physical model and completely satisfies the demands of classifiers. The recognition results are quite acceptable and have validated the reliability of the proposed system.

## 7. Conclusions

In this work, we proposed a novel model-based driving behavior recognition system using motion sensors. The physical model built and data change rule deduced promise the system a good performance with an average accuracy of 93.25% in classifying all 14 types of driving behaviors acquired in real traffic conditions. In spite of different cars and drivers, the proposed system can overcome these differences and performs well universally.

Firstly, based on the related knowledge of rigid body kinematics, we built a physical model to depict car motion on roads and then derived the change rule of data in motion sensors. The established physical model provides the whole system with a theoretical foundation and the data change rule reveals the great difference among different driving behaviors, which provides a clear direction for the following data processing; Secondly, we built the database with 20 h and 1200 km driving in real traffic conditions; Thirdly, having analyzed the main components of noise existing in the data, we utilize the Kalman filter to remove noise. Compared with the conventional low pass filter, the Kalman filter is more in line with the physical model. Then, based on the derived data change rule, we proposed a novel method utilizing the slope, waveform and energy information to extract valid data from the whole driving process. By removing unnecessary data, the memory space of motion sensors can be saved to a great extent. Also, the whole process is automatic, with manual work being avoided.

After the valid data extraction stage, guided by the data change rule, we effectively extracted the data features reflecting the difference among driving behaviors. Compared with the dimensionality reduction methods or the empirical selection of feature sets, our approach of features selection possesses a definite direction and uses less computational resources, which makes the whole system concise and efficient. In the end, we utilized seven distinct classifiers to classify and recognize driving behaviors. An accuracy of 93.25% is achieved by SVM, which is the best among the work in the area of driving behavior recognition. According to the data change rule and real traffic flow, we analyzed the performance of classifiers and the reasons why some samples are classified improperly. Under the guidance of the proposed physical model and data change rule, a driving behavior recognition system was basically established.

In conclusion, our work is very convincing and the proposed system is totally feasible.

In future work, we will extend our network on a large scale and establish a more complete and comprehensive database to provide better data support for the recognition system. In addition, there still exists much room for optimization of features extraction. Therefore, other combinations of features contributing to the recognition accuracy need to be explored. What is more, from the established physical model we can see the potential to recognize more driving behaviors, such as climbing or descending slopes. The recognition of these behaviors will make our system more integrated. Without being restricted by the motion sensors only, we are currently developing a specific module which carries the driving behavior recognition system and can be integrated into other electronic products, such as automobile data recorders and Bluetooth products. A more comprehensive driving evaluation mechanism will be established in the future.

## Figures and Tables

**Table 1:** Information about cars used in the experiment.

Year	Manufacturer	Model	Vehicle Type	Engine
2012	Hyundai	Sonata	Sedan	2.0 L L4
2011	Honda	Accord	Sedan	2.0 L L4
2012	Toyota	Camry	Sedan	2.0 L L4
2013	Nisan	Teana	Sedan	2.0 L L4
2014	Volvo	XC60	SUV	2.0 T L4

**Table 2:** The behaviors classified by the proposed system.

Normal Behaviors	Aggressive Behaviors
Acceleration (ACC)	Aggressive Acceleration (A-ACC)
Brake (Brake)	Aggressive Brake (A-Brake)
Left turn (LT)	Aggressive Left turn (A-LT)
Right turn (RT)	Aggressive Right turn (A-RT)
U turn (UT)	Aggressive U turn (A-UT)
Left lane change (LLC)	Aggressive Left lane change (A-LLC)
Right lane change (RLC)	Aggressive Right lane change (A-RLC)

**Table 3:** Some driving behaviors which can be classified even by the information of a single axis.

Data Axis Utilized	Driving Behaviors Can Be Classified
$a_{x}$	ACC and Brake
$a_{y}$ or $\omega_{z}$	LLC and RLC
$a_{y}$ or $\omega_{z}$	LT and RT
$m_{x}$ or $m_{y}$	LT/RT and UT

**Table 4:** The features extracted from the valid data.

Feature	Description
max	Maximum value of the valid data
min	Minimum value of the valid data
avg	Mean value of the valid data
std	Standard deviation of the valid data
peak	The quantity of peak value
pvam	The rate of data which exceeds the mean value
mad	Mean absolute deviation of the valid data
eng	Energy of the valid data
cov	Covariance matrix of the valid data
k	Slope between the maximum and minimum
t	The duration time of the valid data

**Table 5:** List of features selected.

		avg	max	min	std	mad	k	pvam	peak	eng	t	cov
**Acceleration**	$a_{x}$	√	√	√	√	√	√	√	√	√	√	Cov
	$a_{y}$	√	√	√	√	√	√	√	√	√		$a_{x}$
	$a_{z}$					√						$a_{y}$
**Angular Velocity**	$w_{x}$					√						Cov
	$w_{y}$					√						$a_{x}$
	$w_{z}$	√	√	√	√	√	√	√	√	√		$w_{z}$
**Magnetic Induction Intensity**	$m_{x}$	√	√	√	√	√	√	√	√	√		Cov
	$m_{y}$	√	√	√	√	√	√	√	√	√		$a_{y}$
	$m_{z}$					√						$w_{z}$

**Table 6:** The quantitative results of different classifiers.

Classifier	SVM	RBF Network	Logistic	BayesNet	C4.5	Naïve Bayes	k-NN
Accuracy	93.25%	85.55%	89.3%	91.1%	84.75%	83.2%	82.95%

**Table 7:** A comparison between this article and other papers.

Driving Behaviors															
	ACC	Brake	LT	RT	UT	LLC	RLC	A-ACC	A-Brake	A-LT	A-RT	A-UT	A-LLC	A-RLC	TOTAL
Our work	90.00%	100.00%	87.20%	92.10%	93.40%	83.80%	88.90%	88.90%	88.90%	100.00%	100.00%	100.00%	100.00%	100.00%	93.25%
Paper [3]			100.00%	82.00%											91.00%
Paper [7]			83.00%	92.00%	77.00%					100.00%		100.00%	83.00%	100.00%	91.00%
Paper [13]	Unkown	Unkown	Unkown	Unkown		Unkown	Unkown								89.00%

**Table 8:** The classification results of specific driving behaviors.

Driving Behaviors														
	ACC	Brake	LT	RT	UT	LLC	RLC	A-ACC	A-Brake	A-LT	A-RT	A-UT	A-LLC	A-RLC
ACC	36	0	3	0	0	0	1	0	0	0	0	0	0	0
Brake	0	47	0	0	0	0	0	0	0	0	0	0	0	0
LT	0	0	150	6	4	12	0	0	0	0	0	0	0	0
RT	4	1	0	176	0	1	9	0	0	0	0	0	0	0
UT	0	0	4	1	71	0	0	0	0	0	0	0	0	0
LLC	0	0	10	2	0	62	0	0	0	0	0	0	0	0
RLC	0	0	0	7	0	2	72	0	0	0	0	0	0	0
A-ACC	0	0	0	0	0	0	0	8	0	0	0	0	0	1
A-Brake	0	0	0	0	0	0	0	1	8	0	0	0	0	0
A-LT	0	0	0	0	0	0	0	0	0	8	0	0	0	0
A-RT	0	0	0	0	0	0	0	0	0	0	9	0	0	0
A-UT	0	0	0	0	0	0	0	0	0	0	0	4	0	0
A-LLC	0	0	0	0	0	0	0	0	0	0	0	0	8	0
A-RLC	0	0	0	0	0	0	0	0	0	0	0	0	0	7

**Table 9:** The recognition rate of specific driving behaviors using SVM algorithm.

Driving Behaviors														
	ACC	Brake	LT	RT	UT	LLC	RLC	A-ACC	A-Brake	A-LT	A-RT	A-UT	A-LLC	A-RLC
Accuracy	90.00%	100.00%	87.20%	92.10%	93.40%	83.80%	88.90%	88.90%	88.90%	100.00%	100.00%	100.00%	100.00%	100.00%
